# Exposure to radiofrequency radiation increases the risk of breast cancer: A systematic review and meta-analysis

**DOI:** 10.3892/etm.2021.9903

**Published:** 2021-03-10

**Authors:** Ya-Wen Shih, Anthony Paul O’Brien, Chin-Sheng Hung, Kee-Hsin Chen, Wen-Hsuan Hou, Hsiu-Ting Tsai

Exp Ther Med 21:Article no. 23, 2021; DOI: 10.3892/etm.2020.9455

Following the publication of the above systematic review and meta-analysis, an interested reader drew to the Editor’s attention that there were potentially a number of concerns associated with the eight source papers on which the study had been based. Two of the studies were considered to have been based on 50 Hz electromagnetic fields and not on radiofrequency radiation; furthermore, one reference used an inaccurate risk estimate for the wrong exposure (shiftwork instead of RF), and one of the studies was focused on male breast cancer (the others all being concerned with female breast cancer).

These issues were drawn to the attention of the authors, who did prepare a rebuttal in response to the reader’s comments. Independently, the Editorial Board also conducted an independent investigation of the reader’s claims, and reached the conclusion that the sheer number of corrections that would have been potentially required meant that it would not have been readily feasible to publish a Corrigendum. Taking all the factors into consideration, the Editor of *Experimental and Therapeutic Medicine* has therefore decided that the article should be retracted from the Journal on account of the number of uncertainties associated with both the way in which the meta-analysis had been performed and the source papers selected for the study. Note that the authors were not in agreement with the retraction of this paper. The Editor apologizes to the readership of the Journal for any inconvenience caused by the retraction of this article.

